# Revealing the Radial Effect on Orientation Discrimination by Manual Reaction Time

**DOI:** 10.3389/fnins.2017.00638

**Published:** 2017-11-24

**Authors:** Lixin Liang, Yang Zhou, Mingsha Zhang, Yujun Pan

**Affiliations:** ^1^Department of Neurology, The First Clinical College of Harbin Medical University, Harbin, China; ^2^State Key Laboratory of Cognitive Neuroscience and Learning, Beijing Normal University, Beijing, China; ^3^Department of Neurobiology, University of Chicago, Chicago, IL, United States

**Keywords:** orientation perception, grating stimuli, visual field, SRC, hemisphere asymmetry

## Abstract

It has been shown that the sensitivity and accuracy of orientation perception in the periphery is significantly better when the orientations are radial with respect to the fixation point than when they are tangential. However, since perception and action may be dissociated, it is unclear whether the perceptual radial effect has a counterpart in reaction time (RT) of motor responses. Furthermore, it is unknown whether or how stimulus-response-compatibility (SRC) effect interacts with the radial effect to determine RT. To address these questions, we measured subjects' manual RT to grating stimuli that appeared across upper visual field (VF). We found that (1) RTs were significantly shorter when a grating was oriented closer to the radial direction than when it was oriented closer to the tangential direction even though the perceptual accuracies for the more radial and more tangential orientations were not significantly different under our experimental condition; (2) This RT version of the radial effect was larger in the left VF than in the right VF; (3) The radial effect and SRC effect interacted with each other to determine the overall RT. These results suggest that the RT radial effect reported here is not a passive reflection of the radial effect in perceptual accuracy, but instead, represents different processing time of radial and tangential orientations along the sensorimotor pathway.

## Introduction

The radial effect refers to the observation that the sensitivity and accuracy of orientation perception in the periphery is significantly better when a stimulus is radially oriented with respect to the fixation point than when it is tangentially oriented (Rovamo et al., 1982; Bennett and Banks, 1991; Westheimer, 2003; Sasaki et al., 2006). The radial effect has also been used to explain the induced effect of vertical disparity (Matthews et al., 2003). Indeed, the anatomical and physiological basis of the radial effect has been revealed previously (Levick and Thibos, 1982; Leventhal and Schall, 1983; Schall et al., 1986; Sasaki et al., 2006).

Although the radial effect is well established, some questions remain open. First, while previous studies focused on comparing the perceptual sensitivity and accuracy between radial and tangential orientations, no study has investigated possible differences in reaction time (RT) of motor responses to these orientations. Since there are studies demonstrating dissociations between perceptual and motor measurements under certain conditions (Goodale and Milner, 1992; Goodale, 2014), a perceptual radial effect does not necessarily imply a RT radial effect. On the other hand, one of the main purposes of sensory processing is to guide motor response. It is therefore interesting to investigate whether the perceptual radial effect has a motor counterpart. Second, previous studies only compared orientation sensitivity and accuracy between radial and tangential orientations at a given location in the VF. It is unclear how the radial effect on orientation perception changes as the orienting direction gradually changes from radial to tangential directions. Third, stimulus-response-compatibility (SRC) effect (also known as Simon effect) also affects RT (Fitts and Seeger, 1953; Simon and Rudell, 1967; Wallace, 1971; Whitaker, 1982; Hommel, 2011; Styrkowiec and Szczepanowski, 2013) and it has not been investigated whether and how it interacts with the radial effect.

To address these questions, we designed an orientation discrimination task in which a grating stimulus (+45° or −45°) appeared randomly at one of nine equally spaced locations in the upper VF. Thus, when the grating appears at one of these nine locations, its direction with respect to the fixation point is radial, tangential or some intermediate orientations. We measured subjects' manual RT for orientation discrimination across the range of radial/tangential conditions. Subjects showed significantly shorter RTs to gratings closer to radial orientation than closer to tangential orientation, and these differences were not attributable to the speed-accuracy tradeoff. Such RT differences decreased as the grating became less radially-oriented and tangentially-oriented. In addition, the magnitude of radial effect on RT was significantly larger in the left VF than in the right VF, which further supports the argument that the spatial perception is asymmetrically processed between left and right hemispheres (Corballis et al., 2002; Boulinguez et al., 2003; Corballis, 2003; Okubo and Nicholls, 2008). Moreover, the radial effect and SRC effect strongly interact with each other to determine the overall RT.

## Materials and methods

**Figure 1:**
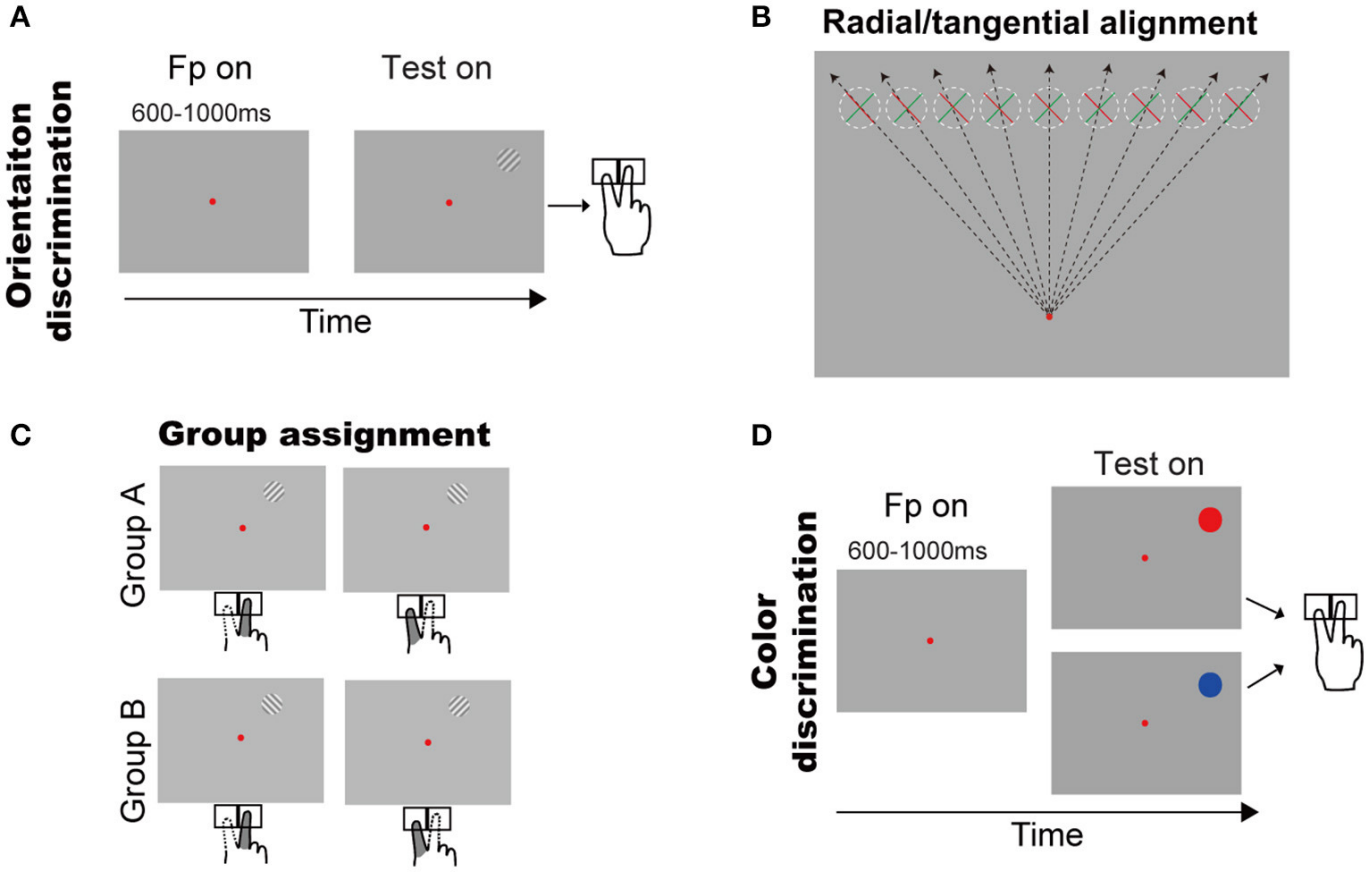
Behavioral tasks and the two groups of subjects. **(A)** Orientation discrimination task. A trial started with a small red fixation point and subjects fixated on it. After a random delay, a grating appeared in upper visual field. Subjects had to press a left or right key to report the grating orientation (see Methods and Results for details). **(B)** Illustration of the grating orientations respect to the fixation point. The dashed circles indicate the grating possible locations across trials and green/red bar indicates grating with +45°/−45°. **(C)** Illustration of finger responses to the gratings in two groups. **(D)** Color discrimination task. A trial started with a small red fixation point and subjects fixated on it. After a random delay, a color patch (red or blue) appeared in upper visual field. Subjects had to press a left or right key to report the color of stimulus.

Eighteen right-handed subjects with normal vision (age 18–28, 17 naïve, 7 male and 11 female) participated in the present experiments. All subjects took part in the first experiment, named as orientation discrimination task (Figure 1A). Before the experiments started, each subject was examined about the self-judged orientation (left or right) to the two gratings (+45° and −45°), by pressing either a left key or a right key as response of his/her judgment. Then, the subjects were induced to remain such self-judged orientation throughout the orientation discrimination experiment. Twelve of eighteen subjects took part in the second experiment, named as color discrimination task (Figure 1D). All experiments followed the guideline of the Institutional Review Board (IRB) of the State Key Laboratory of Cognitive Neuroscience and Learning, Beijing Normal University. The protocol was approved by the same committee. All subjects gave written informed consent in accordance with the Declaration of Helsinki.

### Experimental setup

All visual stimuli were presented on an LCD monitor (BENQ XL2720Z, 1,920 × 1,080 pixels, 100 Hz vertical refresh rate) calibrated and linearized with a Konica Minolta LS-110 photometer. The subjects sat in front of the monitor at a viewing distance of 57.5 cm and with their head restrained on a chin rest. Their eye positions were monitored with an infrared image eye tracker (EyeLink 2000 Desktop Mount, SR Research). Two key buttons were placed in front of the subjects. We used MATLAB (Mathworks) with Psychtoolbox extension (Brainard, 1997; Pelli, 1997) on a PC to present stimuli and collect the manual RT data.

### Behavioral tasks

#### Orientation discrimination task (Figure 1A)

A trial began with a red fixation point (16.0 ± 0.2 cd/cm2) appearing in the center of the screen (14.4 ± 0.2 cd/cm^2^). The subjects had to initiate fixation after the fixation onset, and were required to maintain fixation throughout each trial. A trial was excluded if the eye position left a circular window (3° radius) centered at the fixation point. After a random delay (600–1,000 ms), a sine grating (10% contrast, 14.4 cd/m^2^ mean luminance, 2° diameter) of either +45° or −45° diagonal grating appeared at one of nine possible locations, which were horizontally aligned with equal space (2°) from 8° left to 8° right of the vertical meridian and were 8° above the fixation point (Figure 1B). Thus, the positive and negative diagonal lines from the fixation point went through the centers of the leftmost and rightmost locations, respectively. Consequently, the +45° or −45° gratings were, respectively, radial and tangential at the rightmost location, and tangential and radial at the leftmost location. When the gratings' location changed from leftmost to rightmost, either of the two gratings will cover some intermediate orientations between radial and tangential. Subjects were instructed to report the self-judged grating orientation (left or right) as fast as possible by pressing a key accordingly (left or right) using the index or middle finger of the right hand, respectively. The inter-trial interval was 2 s.

#### Color discrimination task (Figure 1D)

The aim of this experiment was to test whether there were intrinsic group differences in sensorimotor transformation. The task sequence was identical to the orientation discrimination task, except that the grating stimulus was replaced by a gray patch (0.8° diameter, 14.3 ± 0.3 cd/m^2^ mean luminance) tinted either red or blue. Subjects were instructed to report the red and blue colors as fast as possible by pressing left and right key, respectively.

#### Stimulus parameters

To reduce performance variations across sessions and subjects, we used a staircase procedure to determine stimulus parameters at threshold performance for each subject at the start of each session (day). For the orientation discrimination task, we varied the spatial frequency of gratings. For the color discrimination task, we varied the R or B index of color patches while keeping the remaining two indices equal to the RGB value of the gray background. For parameters determination, we fixed the duration of stimulus appearance (30 ms for gratings and 50 ms for color patches) and found thresholds at 78% correct performance by fitting cumulative Gaussian function to psychometric curves. Across all subjects and sessions, the threshold of spatial frequency ranged from 3.8 cycle/degree to 5.6 cycle/degree, and the threshold of color- index ranged from 1 to 5%.

### Data analysis

#### RT calculation

The RT was calculated by using the same criteria as reported previously (Zhou et al., 2012, 2017). Briefly, we collected a total of 41,787 trials and excluded 6.18% of them in which subjects broke fixation or reported incorrectly, or the RTs differed from the mean by more than 3 standard deviations. For each subject, we calculated the mean of raw RT as well as the mean of normalized (differential) RT due to the fact of the existence of RT variation among subjects. Such RT variation may influence the results of statistical analysis.

To calculate differential RT, we used both subtraction and division models. First, we set RTs responding to the grating (+45° and −45°) or patch (red and blue) appeared at the vertical meridian (straight ahead) as baseline condition, and then for each subject subtract/divide the mean RT at baseline condition from the mean RT at the other locations under same stimulus condition, respectively. Since the orientations of two gratings (+45° and −45°) at the vertical meridian location are in the middle between radial and tangential directions and there is no spatial SRC effect, the differential RTs predominately reflect the influence of radial effect on the temporal process of orientation discrimination.

#### Response accuracy calculation

We calculated each subject's response accuracy (correct ratio) for each grating orientation (+45° and −45°) at each tested location.

#### SRC effect quantification

Although the spatial locations of the grating were task-irrelevant, trials could be separated into SRC-compatible and SRC-incompatible conditions, based on the spatial relationship between the location of the grating and the direction of response key (finger). The SRC-compatible condition included trials in which the locations of grating and the response key were spatially compatible (e.g., gratings that appeared in the left VF were associated with left-key responses; gratings that appeared in the right VF were associated with right-key responses). In contrast, the SRC-incompatible condition included trials in which the grating and the response were spatially opposite (e.g., the gratings that appeared in the left VF were associated with right-key responses; gratings that appeared in the right VF were associated with left-key responses).

#### Radial effect quantification

Considering the fact that subjects' responses are highly influenced by the SRC effect (also known as the Simon effect) (Simon and Rudell, 1967; Wallace, 1971; Whitaker, 1982; Hommel, 2011; Styrkowiec and Szczepanowski, 2013), which results in the faster and more accurate responses in SRC compatible conditions than in SRC incompatible conditions. Thus, to quantify the radial effect on RT, we need to exclude the influence of SRC. To do so, subjects were separated into two groups based on their subjective preference to the same grating as titled to left or right (Figure 1C). Group A contained 7 subjects who preferred the right-key response for the +45° grating and the left-key response for the −45° grating. Group B contained 11 subjects with the opposite preferences. Therefore, we created experimental conditions in which the SRC conditions were same for group A and group B subjects (same stimulus location and same key-press response), but the orientations of the gratings were opposite, with one being closer to the radial orientation and the other being closer to the tangential orientation (Figure 1B). The RT difference between two groups of subjects under the same SRC conditions predominately reflects the radial effect.

#### Magnitude of radial effect

We defined the value of RT difference between two groups of subjects at each tested location as the magnitude of radial effect on orientation discrimination at this location. The magnitude of radial effect was calculated by comparing the absolute average RT difference between group A subjects and group B subjects.

## Results

The information of involvement of each subject in the experiments and the mean of raw RTs under different visual stimulation conditions (left VF vs. right VF) in orientation and color discrimination tasks are shown in Tables 1, 2, respectively. However, considering the fact that the large variation of RTs among individual subjects might affect the results of some statistical analyses, we calculated the differential RTs (see methods for details) for each subject and further used them in various statistical analyses. Since subtraction and division models showed consistent results, only data of differential RTs from subtraction model were shown thereafter.

**Table 1:** The mean RTs under different conditions in orientation discrimination task.

	**RT (ms)**					**RT (ms)**			
**Subject**	**Left-key**		**Right-key**		**Subject**	**Left-key**		**Right-key**	
**Group A**	**LVF**	**RVF**	**LVF**	**RVF**	**Group B**	**LVF**	**RVF**	**LVF**	**RVF**
CY	567.3	594	571.5	563.5	GQ	622.3	585.4	648.3	614.4
DY	493	590.1	631.8	508.4	LLX	600	495.6	521.7	507
HZY	561.4	585	638	562.7	LD	620.8	579.6	636.2	592.9
LYY	469.6	506.6	560.5	500.2	LY	509.3	477.7	485.5	484
PY	555.7	568.3	617.1	495	LSK	487.7	458.7	498.3	491.9
YL	474.6	580.2	666.5	479.9	RSF	571.6	570.8	567.9	547.9
YYH	455.7	518.5	578.6	473.8	SJ	599.2	552.6	556.9	599.2
					TRM	581.5	531.8	562.9	562.5
					WYQ	460.2	446	455.3	426.5
					WZY	424.2	421.1	442.9	407.8
					XC	488.2	443.2	479.7	516.9

**Table 2:** The mean RTs under different conditions in color discrimination task.

	**RT (ms)**					**RT (ms)**			
**Subject**	**Left-key**		**Right-key**		**Subject**	**Left-key**		**Right-key**	
**Group A**	**LVF**	**RVF**	**LVF**	**RVF**	**Group B**	**LVF**	**RVF**	**LVF**	**RVF**
CY	698.8	709	687.5	711.1	LLX	448.9	456.6	486.4	445.8
HZY	557	573.7	584	566.5	LD	637	691.2	695.4	657.2
LYY	495.9	529.2	541.2	533.3	LSK	472.6	476.6	520.4	495.4
YL	696.3	697.3	698.5	662.4	RSF	603.7	610.7	582.4	580.6
YYH	588.5	617.8	580.9	564.2	SJ	609.1	627.1	601.1	593.7
					TRM	592.6	604.7	675.4	650.4
					WZY	389.3	404.9	405.9	405.2

### Reaction times (RTs) for orientation discrimination were significantly shorter when a grating was oriented closer to the radial direction than when it was oriented closer to the tangential direction

We employed the three-way ANOVA analysis [two key responses (left, right) × two gratings (+45°, −45°) × nine locations (−8°, −6°, −4°, −2°, 0°, 2°, 4°, 6°, 8°)] to test the significance of radial effect on RTs in the orientation discrimination task. The results of ANOVA analysis of the differential RTs showed a significant interaction between the grating orientations and grating locations [*F*_(8, 216)_ = 31.8, *p* = 1.42e-35], and a significant interaction between the key-responses and grating locations [*F*_(8, 216)_ = 11.5, *p* = 3.28e-14].

**Figure 2:**
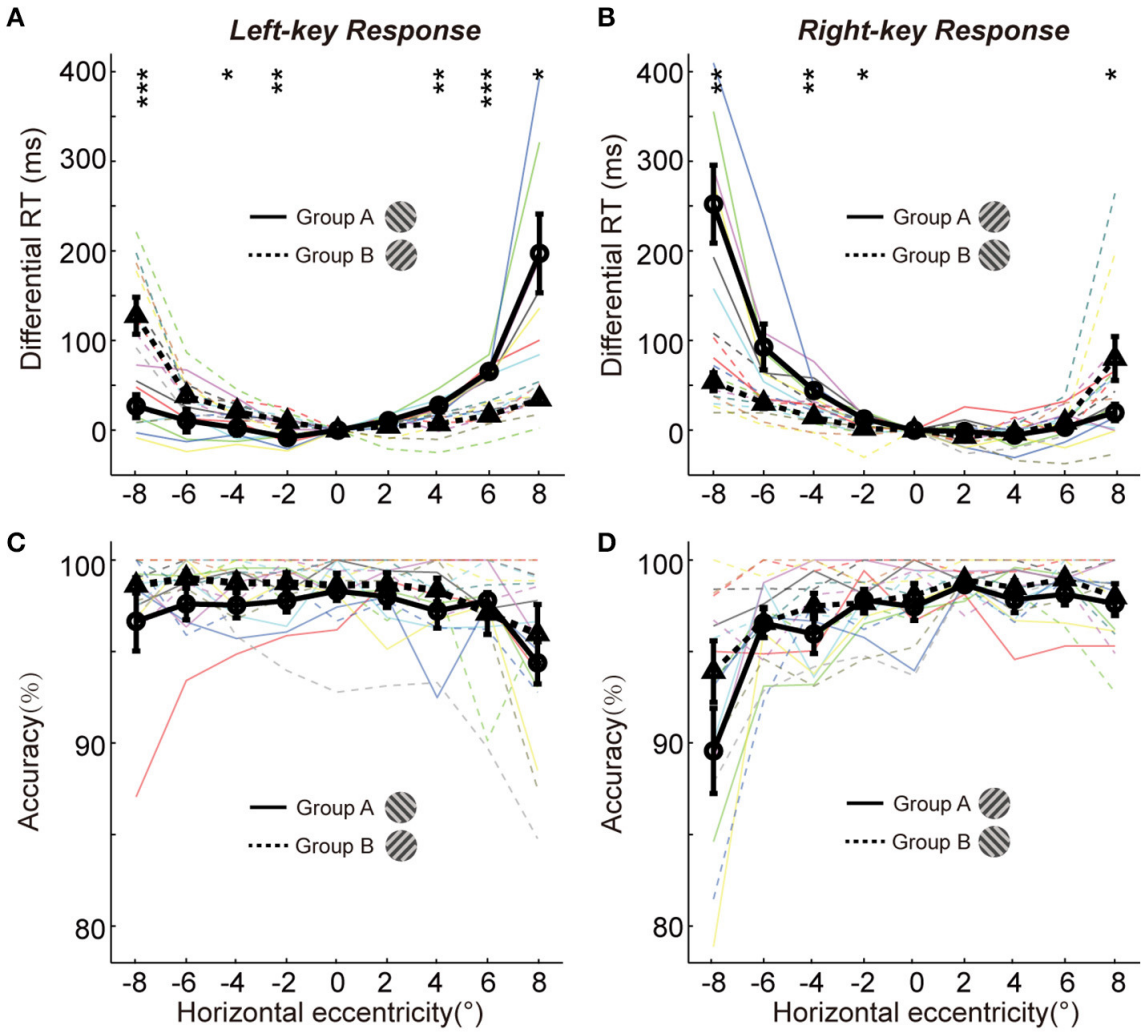
The differential RTs and accuracies of two groups in orientation discrimination task. **(A,B)** The differential RTs are plotted against the grating horizontal eccentricity with left-key **(A)** and right-key **(B)** response. **(C,D)** The accuracies are plotted against the grating horizontal eccentricity with left-key **(C)** and right-key **(D)** response. The solid black curve (through circles) and dashed black curve (through triangles) represent mean differential RTs/accuracies of group A and B, respectively. The black vertical bars denote ±SEM. The thin solid and dash color lines represent the differential RTs/accuracies of individual subject in group A and B, respectively. Asterisk denotes the results of *post-hoc t*-test between two groups: ^*^P < 0.05, ^**^P < 0.01, ^***^P < 0.001.

To show more detailed results of radial effect on RTs, the data of differential RTs of two groups are arranged based on the key-response (manual response), i.e., pressing the same key but responding to the opposite gratings (+45° vs. −45°) in Figures 2A,B, respectively. Although the manual response is the same (Figure 2A, left-key response; Figure 2B, right-key response) and the spatial location of grating is the same (x-axis showing the horizontal eccentricities of grating), the distribution pattern of the differential RTs is very different between the two groups of subjects. Such difference could not be explained by the SRC effect, because the SRC conditions are identical between two groups of subjects in both Figures 2A,B. Thus, a reasonable explanation is that the differences in differential RT between two groups of subjects are mainly caused by the orientation of gratings (+45° vs. −45°). The differential RTs were shorter when the gratings were oriented closer to the radial direction than when they were oriented closer to the tangential direction in both left (solid line relative to dashed line) and right (dashed line relative to solid line) VFs.

We further performed the *post-hoc t*-tests at each stimulated location to test the differential RT difference between two groups of subjects. For left-key response (Figure 2A, groups A subjects responding to the −45° grating; group B subjects responding to +45° grating), in the left VF the RTs of the group A subjects were significantly shorter at 3 of 4 locations [*t*-test, maximum *p* = 0.046, *t*_(16)_ = −2.3] than that of the group B subjects; in contrast, in the right VF the RTs of the group B subjects were significantly shorter than that of the group A subjects at 2 of 4 locations [*t*-test, maximum *p* = 0.010, *t*_(16)_ = 3.7].

Similar results were observed from the RTs of right-key response (Figure 2B, groups A subjects responding to the +45° grating; group B subjects responding to −45° grating). In the left VF, the RTs of the group B subjects were significantly shorter at 3 of 4 locations [*t*-test, maximum *p* = 0.042, *t*_(16)_ = 2.2] than that of the group A subjects. In contrast, in the right VF, the RTs of the group A subjects were significantly shorter than that of the group B subjects at 1 of 4 locations [*t*-test, *p* = 0.010, *t*_(16)_ = −2.3].

Overall, the differential RTs were significantly shorter when the grating orientation was closer to the radial direction than when it was closer to the tangential direction.

### The radial effect on RT was not due to speed-accuracy trade-off (SATO)

It is commonly known that the speed of object discrimination could become faster by reducing the response accuracy, which is known as the SATO. To examine whether the radial effect on RT was due to SATO, we employed a three-way ANOVA [two key responses (left, right) × two gratings (+45°, −45°) × nine locations (−8°, −6°, −4°, −2°, 0°, 2°, 4°, 6°, 8°)] to analyze the variance of response accuracy between different experimental conditions. The results showed no significant interaction either between grating orientations and grating locations [*F*_(8, 216)_ = 1.4, *p* = 0.18] or between key responses and grating locations [*F*_(8, 216)_ = 1.4, *p* = 0.18]. Also, the *post-hoc t*-test at each location showed no significant difference in response accuracy between two groups of subjects in left-key response [Figure 2C, in left VF, minimum *p* = 0.17, *t*_(16)_ = −1.5; in right VF, minimum *p* = 0.36, *t*_(16)_ = −1.0]. Similar results were observed in right-key response condition [Figure 2D, in left VF, minimum *p* = 0.16, *t*_(16)_ = −1.5; in right VF, minimum *p* = 0.25, *t*_(16)_ = −1.2]. These results indicate that the radial effect on the temporal process of orientation discrimination is not due to SATO.

### The radial effect on RT in the left VF was stronger than that in the right VF

To test whether the radial effect on RT is equally distributed in the left and right VFs, we performed a four-way ANOVA [two key responses (left, right) × two gratings (+45°, −45°) × four eccentricities (2°, 4°, 6°, 8°) × two VFs (left, right)]. The results showed significant interaction among grating × eccentricity × VF for differential RTs [*F*_(3, 192)_ = 38.1, *p* = 2.14e-20], which indicated the radial effect on RT is asymmetrically distributed between the left and right VFs. To further explore the spatial characteristics of radial effect on RT across VF, we estimated the magnitude of radial effect at each tested location by calculating the absolute RT difference between two groups of subjects under the same key response conditions.

**Figure 3:**
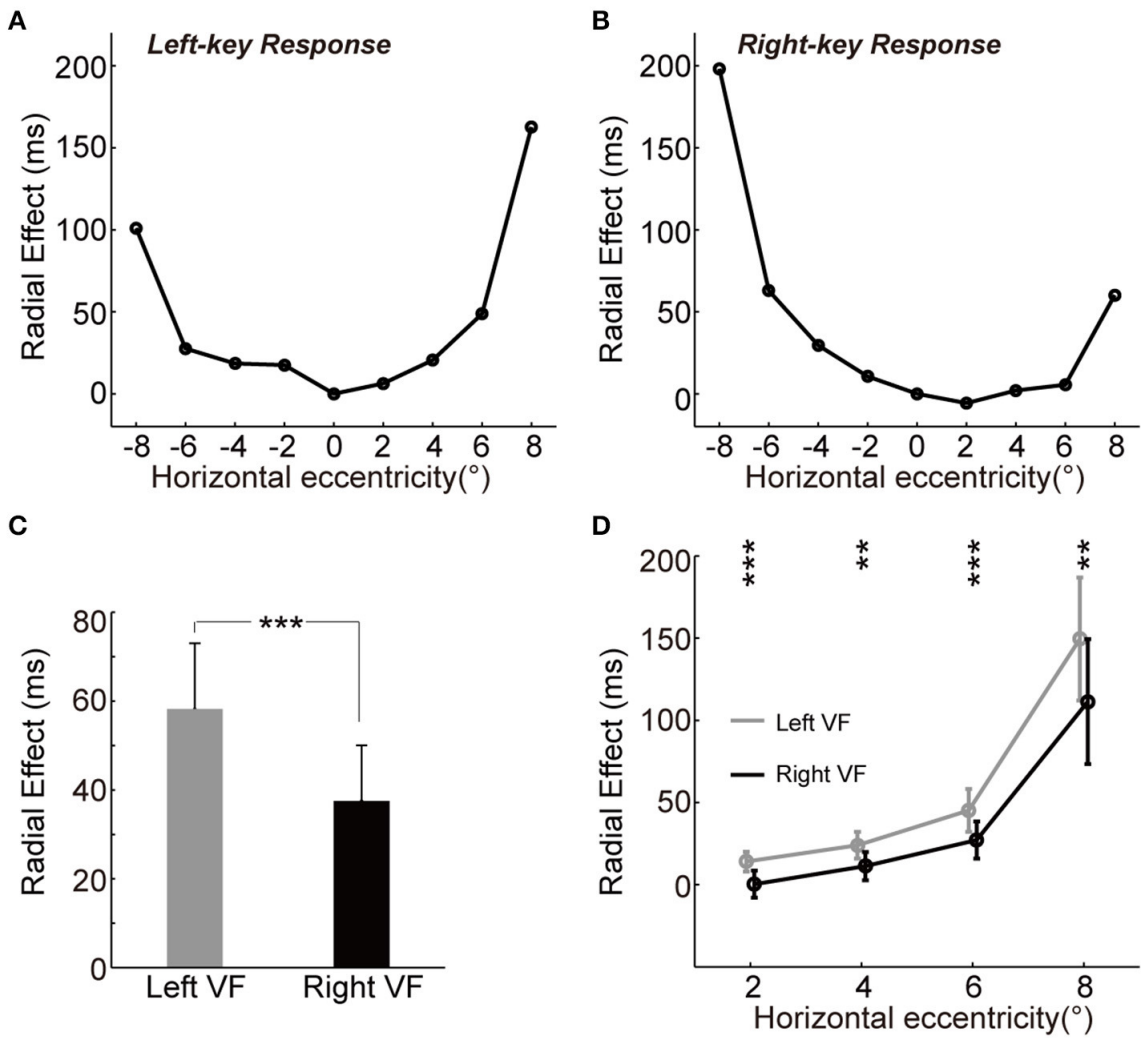
The asymmetry of radial effect. **(A,B)** Tuning curve of the radial effect. The curve (through circles) represents the average of RT differences between two groups with left-key **(A)** and right-key **(B)** responses. **(C)** The radial effect between left and right VFs. **(D)** The radial effect between left and right VFs across each tested horizontal eccentricity. The gray curve (through gray circles) and black curve (through black circles), respectively represent the average of the radial effect in left and right VFs across each tested horizontal eccentricity. All the error bars denote ±SD. Asterisk denotes the results of paired *t*-test: ^**^p < 0.01; ^***^P < 0.001.

We observed the “unbalanced U-shape” tuning curves of the magnitude of radial effect for both left-key and right-key response conditions (Figures 3A,B). The magnitude of radial effect was largest when the grating was either fully radial-oriented or fully tangential-oriented (the leftmost and rightmost locations) and became smaller as the grating became less radial-oriented and less tangential-oriented (closer to the vertical meridian). In addition, the radial effect tuning curve also differed between the left and right VFs in both left-key and right-key response conditions (Figures 3A,B). One possible reason is that the unbalanced tuning curve was, at least partially, due to the influence of SRC effect, i.e., shorter RTs in SRC compatible conditions and longer RTs in SRC incompatible conditions. To simply exclude the SRC effect on the unbalanced tuning curve, we mixed the radial effect of left-key (Figure 3A) and right-key (Figure 3B) response together correlated with the location of grating stimulation, and then the mean radial effect was calculated for each tested eccentric location. In average, the mixed radial effect was larger in the left VF than in the right VF (Figure 3C, the error bars will be described in next paragraph). The paired *t*-test of mixed radial effect between 4 tested locations in left VF and other 4 locations in right VF showed significant difference [*t*_(3)_ = 3.5, *p* = 0.040]. However, considering the fact that the few number of samples (only four paired eccentric locations were tested between left and right VFs, respectively) might strongly bias the results of *t*-test, we used the following method to confirm the above result.

For each key-press condition, we first calculated the mean differential RT of group A subject (*n* = 7) at each tested location, and then calculated the absolute difference (radial effect) between this mean differential RT and differential RT of each group B subject (*n* = 11) at the same tested location. Thus, we created a 9 (locations) × 11 (subjects) matrix of data set for each key-press condition, respectively. To compare the radial effect between left and right VFs, these two 9 × 11 matrices were mixed together based on the eccentric location of grating stimulation, and then the mean radial effect of each subject (*n* = 11) at each tested eccentric location was calculated. Paired *t*-test of this mixed 9 × 11 matrix showed significant larger radial effect in the left VF than in the right VF [*t*_(10)_ = 5.6, *p* = 2.25e-4]. Error bars in Figure 3C represents the mean standard deviation of these 11 subjects.

Such asymmetric distribution of the radial effect was further confirmed by comparing the differential RT difference (radial effect) between mirror locations relative to the vertical meridian (Figure 3D), in which the mean radial effect with standard deviation at each tested eccentric location was calculated from the mixed 9 × 11 matrix. Paired *t*-test showed that the radial effect was stronger in the left VF than in the right VF at all tested off-middle locations [maximum *p* = 0.0035, *t*_(10)_ = 3.8].

### The radial effect and SRC effect interact to determine RT

A line of studies has found that the behavioral performance is faster and more accurate in SRC-compatible conditions than in SRC-incompatible conditions (Fitts and Seeger, 1953; Wallace, 1971; Whitaker, 1982), a finding also known as the Simon effect (Simon and Rudell, 1967; Hommel, 2011; Styrkowiec and Szczepanowski, 2013). We have shown earlier that the SRC effect strongly modulated the radial effect (Figures 2A,B). Here, we employed a three-way ANOVA analysis [two key responses (left, right) × two gratings (+45°, −45°) × nine locations (−8°, −6°, −4°, −2°, 0°, 2°, 4°, 6°, 8°)] to statistically test the interaction between radial effect and SRC effect. The results showed that the interaction between radial effect and SRC (key × grating × location) was significant [*F*_(8, 216)_ = 2.7, *p* = 0.0069].

**Figure 4:**
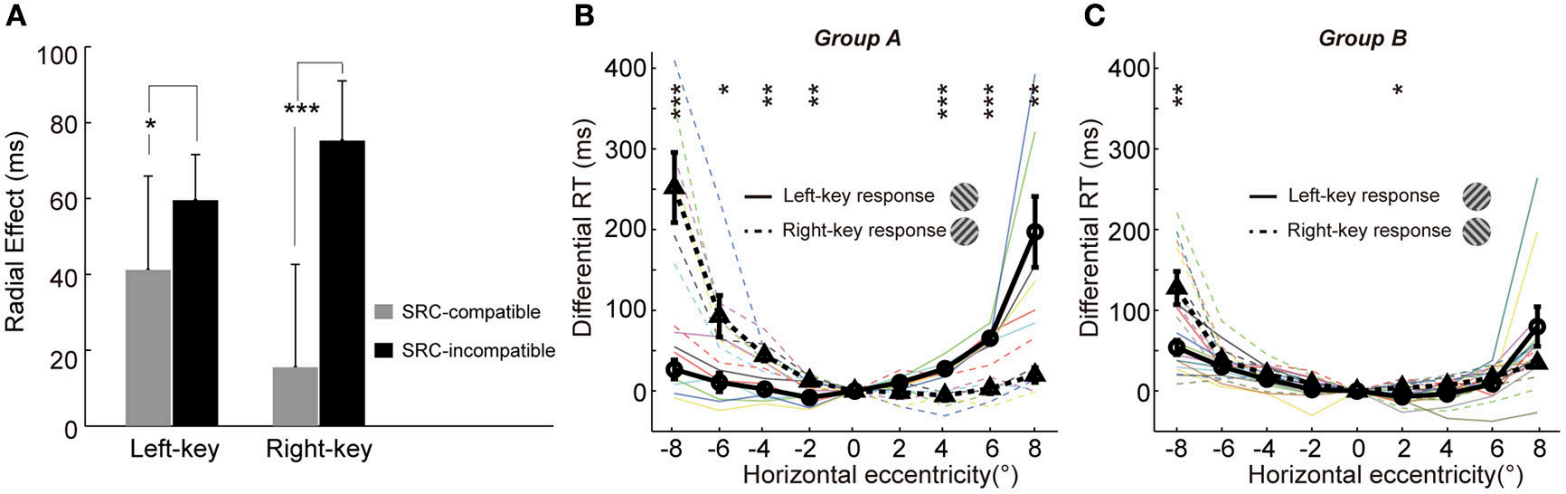
Mutual interaction between the radial effect and SRC effect. **(A)** The influence of SRC effect on the radial effect. The radial effect is shown in the SRC-compatible (gray) and SRC-incompatible (black) conditions with left-key and right-key response, respectively. The error bars denote *SD*. **(B,C)** The influence of the radial effect on SRC effect. The differential RT is plotted against the grating horizontal eccentricity in group A **(B)** and B **(C)**. The solid black curve (through circles) and dashed black curve (through triangles) represent mean differential RTs with left-key and right-key responses, respectively. The black vertical bars denote ±SEM. The thin solid and dash color lines represent the differential RTs of individual subject with left-key and right-key responses, respectively. Asterisk denotes the results of *post-hoc* paired *t*-tests between left-key and right-key responses in each group: ^*^P < 0.05, ^**^P < 0.01, ^***^P < 0.001.

Furthermore, the influence of SRC to radial effect was very different between the two SRC conditions, as shown in Figure 4A (the averaged radial effect of left and right 4 off-middle locations in Figures 3A,B, respectively). The averaged radial effect was greater in the SRC-incompatible condition than in the SRC-compatible condition in both left and right key responses. Two sample *t*-test resulted the significant difference in radial effect between two SRC conditions [left key response, *t*_(10)_ = −2.4, *p* = 0.037; right key response, *t*_(10)_ = 6.6, *p* = 5.81e-05].

On the other hand, the radial effect strongly affected the SRC effect too. Based on the relationship between the radial effect and SRC effect, trials could be separated into two types (Figure 1C): radial effect and SRC effect matched trials (group A: radially-oriented conditions were associated with SRC-compatible conditions and tangentially-oriented conditions were associated with SRC-incompatible conditions), and radial effect and SRC effect non-matched trials (group B: radial-oriented conditions were associated with SRC-incompatible conditions and tangential-oriented condition were associated with SRC-compatible condition). Although the SRC conditions were same, the distributions of the differential RTs were dramatically different between group A subjects (radial effect and SRC effect match, Figure 4B) and group B subjects (radial effect and SRC non-match, Figure 4C). While differential RTs of group A subjects showed clear SRC effect (circles are smaller than triangles in the left VF and vice versa in the right VF, Figure 4B), differential RTs of group B show dramatically diminished SRC effect (Figure 4C). Two sample *t*-test confirmed that the differential RT differences in group A subjects were significantly greater than in group B subjects [*t*_(16)_ = 5.0, *p* = 0.0010].

Thus, these results clearly showed that radial effect and SRC effect interact with each other to determine the overall RTs.

### The RT differences in orientation discrimination task between two groups were not caused by subjects' difference in the sensorimotor transformation

In the present study, the radial effect was quantified by calculating the RT differences between two groups. One intuitive argument is that the observed RT differences might be caused by intrinsic group differences in the sensorimotor process. Although this is very unlikely, we nevertheless performed a control experiment to test it.

**Figure 5:**
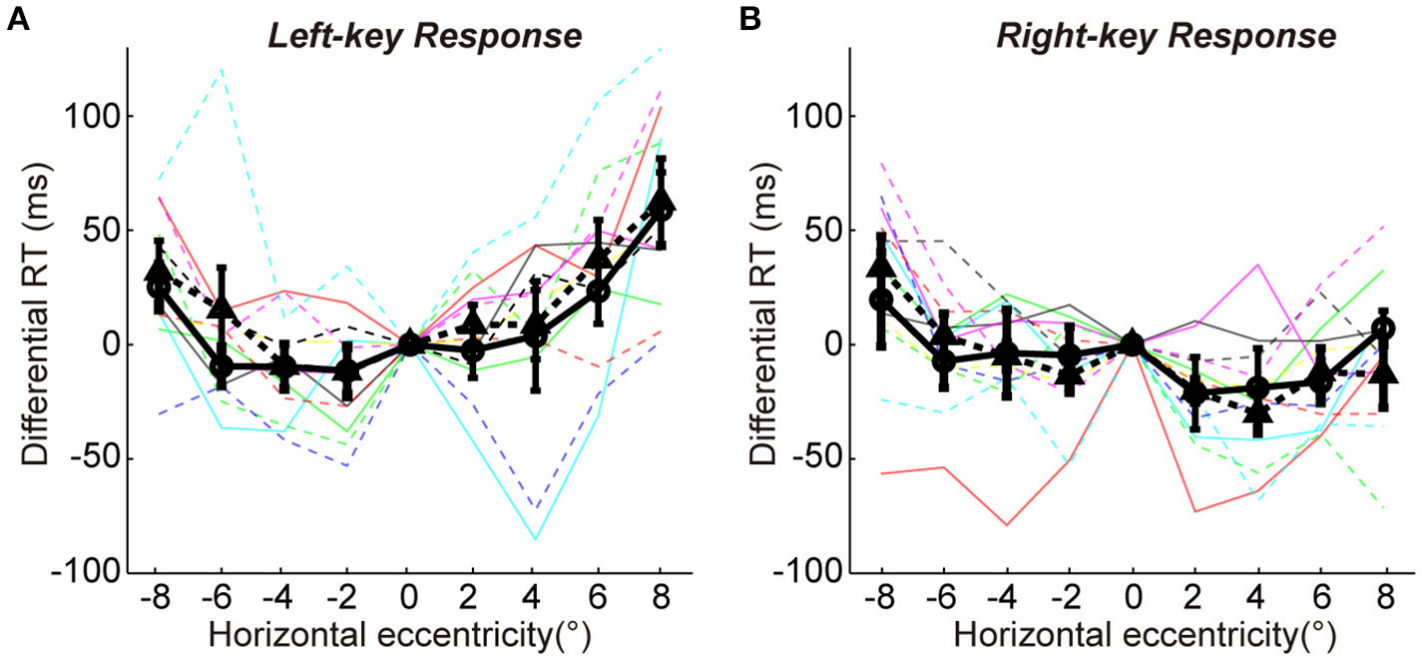
The differential RTs in color discrimination task. The differential RT is plotted against the color patch horizontal eccentricity with left-key **(A)** and right-key **(B)** response. The solid black curve (through circles) and dashed black curve (through triangles) represent mean differential RTs with left-key and right-key responses, respectively. The black vertical bars denote ±SEM. The thin solid and dash color lines represent the differential RTs of individual subject with left-key and right-key responses, respectively.

We employed a color discrimination task to test the similarity of sensorimotor process between two groups. In this task, the test stimuli and response strategy were exactly same for two groups of subjects (see details in the Methods). First, SRC effect was confirmed by a two-way ANOVA analysis [two key responses (left, right) × two VFs (left, right)]. The results showed a significant interaction between key response and VF for the differential RTs [*F*_(1, 11)_ = 32.0, *p* = 1.47e-04]. Second, the differential RTs were very similar between two groups for both left-key (Figure 5A) and right-key (Figure 5B) responses [left-key response: one-way ANOVA, 2 groups, *F*_(1, 8)_ = 0.4, *p* = 0.53; right-key response: one-way ANOVA, 2 groups, *F*_(1, 8)_ = 0.0, *p* = 0.89]. Also, the differences of differential RTs in all tested off-middle locations did not reach the significant level [*t*-test, minimum *p* = 0.25, *t*_(10)_ = −1.2]. Thus, the difference in differential RTs between the two groups in orientation discrimination task was not due to the differences in sensorimotor process between subjects.

## Discussion

Orientation processing is a key element of visual perception. Despite the intuitive view that orientation processing is isotropic, it has been reported that human subjects exhibit perceptual differences for different orientations, including the oblique effect (Appelle, 1972; Berkley et al., 1975), the cardinal effect (Rijsdijk et al., 1980; Carrasco et al., 2001), and the radial effect (Rovamo et al., 1982; Bennett and Banks, 1991; Westheimer, 2003; Sasaki et al., 2006). Different from previous radial effect studies, which focused on comparing orientation sensitivity and accuracy between radial and tangential orientations, we systematically assessed the radial effect in term of the processing time along the sensorimotor pathway by measuring the manual RT to a +45° or −45° grating. The grating appeared randomly at one of nine equally spaced locations in the upper VF so that its orientation with respect to the fixation point is radial, tangential or somewhere in-between (Figure 1B). We found significantly shorter RTs to gratings closer to radial orientation than those closer to tangential orientation. Although this RT radial effect was consistent with the previously reported perceptual radial effect (Rovamo et al., 1982; Bennett and Banks, 1991; Westheimer, 2003; Sasaki et al., 2006), the former is not a passive reflection of the latter because under our experimental condition, there was no significant difference in perceptual accuracy between the more radial and more tangential orientations.

When studying the radial effect, one must control for potential confound from another well-known phenomenon, the oblique effect, according to which the perception of cardinal orientations is superior to that of oblique orientations (Appelle, 1972; Berkley et al., 1975; Furmanski and Engel, 2000). Because of the oblique effect, we cannot assess the radial effect by comparing different orientations that include both more cardinal and more oblique orientations at a given location in the VF. Instead, we only used gratings of +45° and −45° orientations which are equally oblique. By presenting them at one of the nine equally spaced locations in the upper VF, we produced more radial and more tangential orientations of various degrees. In this way, we were able to measure the radial effect by comparing the two equally oblique orientations and avoid the confound of the oblique effect.

A byproduct of the present study is that the RT radial effect is asymmetric between the left and right VFs: the effect is larger in the left VF than in the right VF (Figures 3C,D). Such results are consistent with findings of many previous studies (Heilman and Van Den Abell, 1979; Sturm et al., 1989; Corballis et al., 2002; Boulinguez et al., 2003; Corballis, 2003; Okubo and Nicholls, 2008; Zhou et al., 2012), which indicates that the right hemisphere of human brain plays a dominant role in processing spatial information than the left hemisphere does. It has been reported that the spatial information is dominantly processed in the left VF by the right hemisphere (e.g., orientation perception), whereas the non-spatial information (e.g., temporal perception) is dominantly processed in the right VF by the left hemisphere (Galati et al., 2010). In contrast, it has been also reported that the process of orientation perception was faster in the right VF than in the left VF when there were competing orienting stimuli appeared in the left and right VFs simultaneously (Carlei and Kerzel, 2017). Indeed, when we put all differential RTs of two groups of subjects together and then separate them to two groups based on the grating's location either in the left or right VF, one-way ANOVA resulted that the effect of VF–RT was significant [*F*_(1, 17)_ = 5.5, *p* = 0.020]. The mean differential RTs were significant faster in the right VF than in the left VF [*post-hoc* paired *t*-test, *t*_(17)_ = 3.8, *p* = 0.0013], which was consistent with the above finding of shorter RT in the right VF than in the left VF.

We found that the radial effect and SRC effect strongly interact with each other to determine RT. The radial effect was significantly larger in the SRC-incompatible condition than in the SRC-compatible condition, in both the left and right VFs (Figure 4A). Additionally, the RT difference between SRC-incompatible and SRC-compatible conditions was larger when the radial and SRC effects were matched than when they were unmatched (Figures 4B,C). Further studies are needed to explore the neural mechanisms underlying the interaction between the two effects.

We measured the radial effect by comparing the RTs between two groups of subjects. It is thus important to rule out systematic differences in sensorimotor transformation between the two groups. To this end, we showed that the two groups of subjects had very similar RTs in a control task on color discrimination (Figure 5).

One may argue that the RT difference among nine tested locations in the present study might be due to the different visual sensitivity, because the nine tested peripheral locations had different eccentricities (Figure 1B). However, our results cannot be explained by the variation of visual sensitivity with eccentricity. As shown in Figures 2A,B, 4B,C, for some subjects, the RTs at several tested locations and in certain trial conditions (SRC-compatible condition and closer to radial orientation) are shorter than the RT in the baseline condition (grating in vertical meridian, with shortest eccentricity). Thus, it is reasonable to predict that the radial effect should be more obvious if the tested locations are distributed with an equal eccentricity. Thus, our results indeed reflect the influence of the radial effect and SRC effect on RT.

Our results indicate that multiple factors interact to determine the overall RT. We summarize our finding with the following simplified model: RT (stimulus, response, location) = Baseline + Radial effect (stimulus, response) + SRC effect (stimulus, response) + interaction (Radial and SRC effects) + VF (location). It would be interesting to investigate the neural mechanisms underlying these factors and their interactions.

## Author contributions

YZ designed the experiments. LL and YZ collected and analyzed the data. MZ and YP supervised the experiments. All authors are involved in writing the manuscript.

### Conflict of interest statement

The authors declare that the research was conducted in the absence of any commercial or financial relationships that could be construed as a potential conflict of interest.
